# Microarray gene expression profiles from mature gonad tissues of Atlantic bluefin tuna, *Thunnus thynnus* in the Gulf of Mexico

**DOI:** 10.1186/1471-2164-13-530

**Published:** 2012-10-05

**Authors:** Luke D Gardner, Nishad Jayasundara, Pedro C Castilho, Barbara Block

**Affiliations:** 1 Biology Department, Hopkins Marine Station, Pacific Grove, Stanford University, California, 93950, USA; 2 Universidade Federal Rural de Pernambuco (UFRPE/UAST), Av. Dom Manoel Medeiros, s/n, Dois Irmãos 52171-900, Recife, PE, Brasil

## Abstract

**Background:**

Bluefin tunas are highly prized pelagic fish species representing a significant economic resource to fisheries throughout the world. Atlantic bluefin tuna (*Thunnus thynnus*) populations have significantly declined due to overexploitation. As a consequence of their value and population decline, *T. thynnus* has been the focus of considerable research effort concerning many aspects of their life history. However, in-depth understanding of *T. thynnus* reproductive biology is still lacking. Knowledge of reproductive physiology is a very important tool for determining effective fisheries and aquaculture management. Transcriptome techniques are proving powerful and provide novel insights into physiological processes. Construction of a microarray from *T. thynnus* ESTs sourced from reproductive tissues has provided an ideal platform to study the reproductive physiology of bluefin tunas. The aim of this investigation was to compare transcription profiles from the ovaries and testes of mature *T. thynnus* to establish sex specific variations underlying their reproductive physiology.

**Results:**

Male and females *T. thynnus* gonad tissues were collected from the wild and histologically staged. Sub-samples of sexually mature tissues were also measured for their mRNA differential expression among the sexes using the custom microarray design BFT 4X44K. A total of 7068 ESTs were assessed for differential expression of which 1273 ESTs were significantly different (p<0.05) with >2 fold change in expression according to sex. Differential expression for 13 of these ESTs was validated with quantitative PCR. These include genes involved in egg envelope formation, hydration, and lipid transport/accumulation more highly expressed in ovaries compared with testis, while genes involved in meiosis, sperm motility and lipid metabolism were more highly expressed in testis compared with ovaries.

**Conclusions:**

This investigation has furthered our knowledge of bluefin tunas reproductive biology by using a contemporary transcriptome approach. Gene expression profiles in *T. thynnus* sexually mature testes and ovaries were characterized with reference to gametogenesis and potential alternative functions. This report is the first application of microarray technology for bluefin tunas and demonstrates the efficacy by which this technique may be used for further characterization of specific biological aspects for this valuable teleost fish.

## Background

Bluefin tunas are highly migratory species that represent a significant economic resource to fisheries globally [1]. Three species have been identified in the Atlantic Ocean (*Thunnus thynnus*), Pacific Ocean (*Thunnus orientalis*) and Southern Ocean (*Thunnus maccoyii*). The Southern and Atlantic bluefin tuna populations have been in significant decline due to overexploitation [2]; the status of the Pacific bluefin is less well known. All three bluefin tuna species have been the subject of recent research efforts to better understand and manage their populations. In recent years, rapid advances in biological techniques for studying highly migratory species has enabled a better understanding of bluefin tuna population structure, oceanic migrations, habitat utilization and genetics [3,4]. Despite these advances, significant questions remain regarding their reproductive dynamics. Aquacultural development of Pacific and Southern bluefin tuna has improved our knowledge of the reproductive biology for these species through the observation of these species in captivity [5].

Atlantic bluefin tuna has been the most challenging of the three bluefin species to understand, primarily due to their complex population structure, the late ages to reproduction in the western population, and logistical challenges of handling the largest of the three bluefin tuna species in captive environments. Currently, at least three populations of bluefin tuna are recognized in the Atlantic and Mediterranean basins [6-9]. Knowledge of the reproductive biology of Atlantic bluefin tuna is still lacking [4] within these discrete populations and is complicated by the extensive mixing population evident from electronic tagging [10]. Evidence suggests that discrete ages to maturity exist among bluefin tuna populations in the Atlantic basin, with Gulf of Mexico spawning populations maturing later than eastern populations, with a year class range showing maturity as early as 8 years of age and a mean closer to 12 years of age for tuna that are spawning in the Gulf of Mexico [11]. In contrast, bluefin tuna in the eastern Mediterranean Sea have been observed to be mature as early as 4 years of age [12], however the mean age to maturity remains unclear due to the presence of western and eastern Mediterranean populations adding complexity to the population biology and fisheries assessments [13].

Knowledge of reproductive biology is a very important tool for effective fisheries and aquaculture management. To date such research has been largely limited to large-scale manifestations of field biology including, spawning season, sex ratio, batch fecundity, gonadosomatic index and gonad histological analysis [14]. Since knowledge of reproductive biology is a critical component of effective fisheries and aquaculture management, further research into the physiological mechanisms underlying Atlantic bluefin tuna reproductive biology is necessary.

Recently, researchers have begun to address the paucity of information in Atlantic bluefin tuna reproductive physiology. Endocrinology studies have measured circulating concentrations of hormones in reference to sex and maturity as well as the effects of hormonal administration on gonads [14-17]. Similarly, Atlantic bluefin tuna gonad reproductive physiology has also been qualitatively investigated at the transcriptome level. Chini et al. [18] sequenced and partially annotated expressed sequence tag (EST) libraries containing 10163 EST from the testis, ovary and liver of mature *Thunnus thynnus*. Similar transcriptome based studies are becoming increasingly prevalent amongst researchers to investigate physiological processes in a number of fish, especially in model organisms or fish with commercial relevance [19-21]. These transcriptome techniques are becoming an established technology for providing novel insights into physiological processes. The availability of 10163 *T. thynnus* ESTs forms a foundation from which the reproductive physiology of bluefin tunas may be further elucidated.

The aim of this investigation was to identify and compare the transcriptomes of the ovaries and testes of mature Atlantic bluefin tuna to establish sex specific variations underlying their reproductive physiology. For this purpose we generated a novel microarray capable of measuring the differential transcriptional expression of 7068 ESTs from *T. thynnus*. The development of the microarray platform in conjunction with examination of gonadal histology and quantitative PCR (QPCR) identified a number of differentially expressed transcripts with particular relevance to the underlying processes of Atlantic bluefin tuna reproductive physiology.

## Results

### Histology

**Figure 1:**
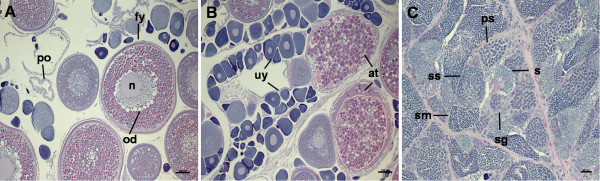
** Histological analysis of *T. thynnus* gonad tissue.** Transverse sections of mature *T. thynnus* gonads at different stages as per Itano et al. [22] and Schaefer [23] designations. (**A**) Stage 6 mature ovaries displaying fully yolked oocytes (fy), oil droplets (od), nucleus (n) and post ovulatory follicles (po), scale bar = 100 μm. (**B**) Stage 4 mature ovaries exhibiting a high proportion of unyolked oocytes (uy) and fully yolked oocytes undergoing atresia (at), scale bar = 100 μm. (**C**) Mature unrestricted spermatogonial testis displaying spermatogonia (sg), spermatocytes (ss), spermatids (sm) and spermatozoa (s), scale bar = 20 μm.

Ovarian tissue from four female Atlantic bluefin tuna from the Gulf of Mexico were examined using histology. These fish ranged in curved fork length from 225 to 266 cm. Representative histological samples of the ovaries are shown in Figure 1. Ovarian tissue from fish 1, 2 and 4 of the four Atlantic bluefin tuna were classed at stage 6 maturity [22] indicated by the presence of fully yolked oocytes, some of which were displaying coalescence of lipid droplets and early stage nucleus migration. Less than 50% of the fully yolked oocytes were undergoing atresia and post ovulatory follicles were also present. According to Itano et al. [22] this is typical of an actively reproductive and spawning fish. Histological observations of the ovarian tissue from fish 3 are more consistent with stage 4 of Itano’s et al. [22] maturity index noting a high incidence of unyolked and partially yolked oocytes. Atresia of fully yolked oocytes was also noted. According to its designation, fish 3 is considered to have reached a fully yolked and potentially reproductive state but has regressed to a reproductively inactive state. 

Testis tissue used in this investigation were obtained from four males ranging in curved fork length from 213 to 269 cm. Histological slide preparations of testis tissue taken from these four males all showed a mature unrestricted spermatogonial type, demonstrated by the presence of all stages of spermatogenesis occurring throughout the seminiferous tubules including spermatogonia, spermatocytes, spermatids and spermatozoa [23].

### Microarray findings

**Figure 2:**
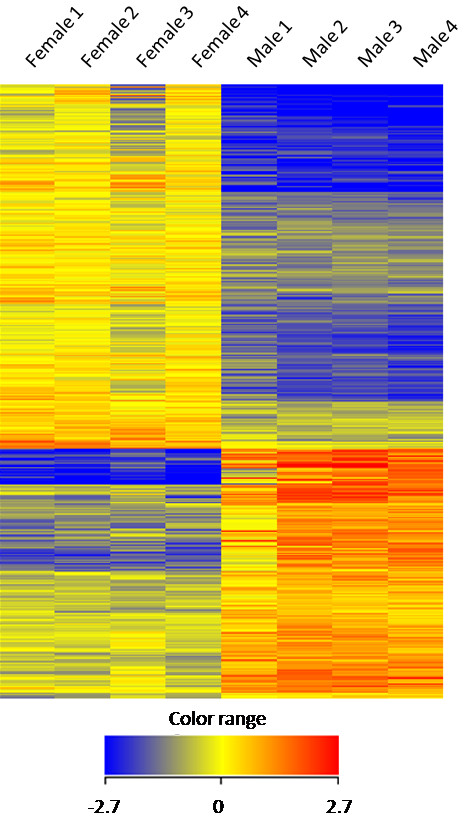
** Hierarchical heat map of *T. thynnus* ESTs differentially expressed among male and female gonad tissue.** A hierarchical clustered heat map showing the log_2_ transformed expression values for microarray features on the BFT 4X44K Array following hybridization of mRNA preparations from *T. thynnus* male and female gonad tissue. The individual features are not labelled but are pictured horizontally showing their relative expression values across all replicates of the male and female gonad tissues. The intensity of the color scheme is calibrated to the log_2_ expression values such that red refers to higher transcript abundance and blue refers to lower transcript abundance. The features displayed are those which are significantly different (p<0.05) between the male and female conditions and have a log_2_ fold change value greater than one.

Gonad tissue from eight wild *T. thynnus* adults caught in the Gulf of Mexico were analysed for differential gene expression on the BFT 4X44K microarray in reference to gender (four males vs. four females). A total of 1820 microarray features were defined as significantly different (p<0.05) with a log_2_ transformed fold change greater than one between the conditions. A hierarchical clustered heat map of these 1820 features across all of the male and female gonads (4 per condition) shows their relative gene expression arranged according to similar feature expression profiles (Figure 2). The upper limits of these features’ log_2_ transformed fold change range from 4.5 in the female gonad specific features to 5.4 in the male gonad specific features. These log_2_ fold change values equate to an absolute fold change of 23 and 43 respectively. Within the 1820 microarray features, 1273 ESTs are represented, 737 of which are reported as having transcript abundances greater than 2-fold higher in the female gonad tissue in comparison to the male gonad tissue (Additional file 1). Likewise, the remaining 536 ESTs were found to be expressed as greater than 2-fold higher in the male gonad tissue in reference to the female gonad tissue (Additional file 2).

Overall, gene expression in males shows a clear concordance among the individuals. The female gene expression profiles also show a high reproducibility among the individuals with the exception of Female 3 (Figure 2). The expression profile of this female gonad sample appears to be partially similar in profile to both the male and female condition. Additional statistical analyses were performed excluding Female 3, however little variation in the list of microarray features deemed statistically significant was noted.

Annotation of the entire BFT 4X44K Array ESTs is relatively low compared to model organisms (zebrafish, killifish, mouse, rat), at 18% of the 7068 ESTs. A further comparison of the annotation percentage between the male and female specific EST lists showed a similarly low level of annotation at 16% for each condition. Considering this level of annotation, automated Gene Ontology annotation categorization techniques were not used to interpret biological meaning for the significant differentially expressed transcripts identified. Instead differentially expressed transcripts were considered individually for biological relevance and grouped accordingly. Gene categories identified from the microarray analysis as pertinent to this study include those concerned with egg envelope formation, yolk proteolysis, oocyte hydration, and lipid accumulation in the case of higher ovarian expression. Relevant categories identified as being expressed in higher concentrations in the testes include meiosis, sperm motility and lipid metabolism.

### Quantitative PCR

**Figure 3:**
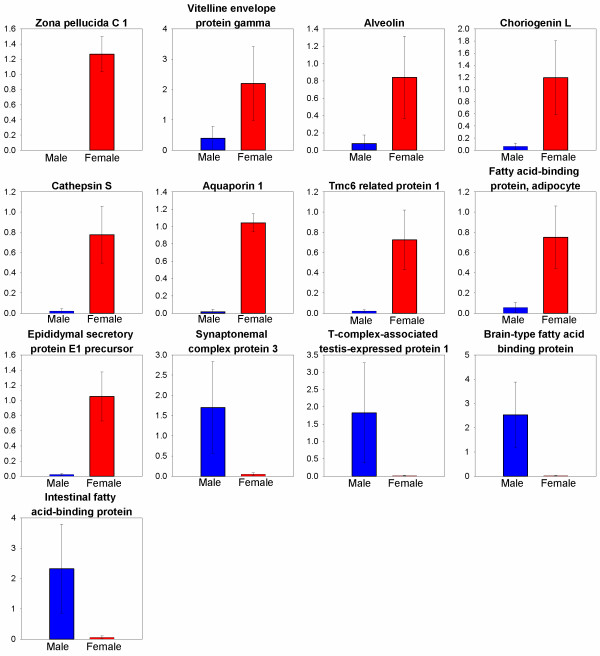
** Mean relative EST abundance between *T. thynnus* male and female gonad tissues as determined by QPCR analysis.** All ESTs graphed are significantly different (p<0.05) with error bars representing standard deviation. Putative EST sequence annotation identities are as follows: Zona pellucida C 1 – TTC00305; Vitelline envelope protein gamma –TTC00056; Alveolin – TTC00935; Choriogenin L – TTC04136; Cathepsin S – TTC00230; Aquaporin 1 – TTC00180; Tmc6 related protein 1 – TTC04625; Fatty acid-binding protein, adipocyte – TTC00964; Epididymal secretory protein E1 precursor – TTC00209; Synaptonemal complex protein 3 – TTC02745; T-complex-associated testis-expressed protein 1 – TTC02749; Brain-type fatty acid binding protein – TTC05153; Intestinal fatty acid-binding protein – TTC05128. Refer to table 1 for accession numbers associated with these ESTs and putative annotations. The colors blue and red refer to male and female gonad tissues respectively. The Y axis represents relative abundance of transcripts between male and female gonads.

*T. thynnus* transcripts shown by microarray analysis to be significantly differentially expressed between male and female gonad tissue were selected for QPCR relative abundance analyses, primarily to validate the microarray differential expression findings. The ESTs selected for QPCR analysis were chosen based on a process favouring ESTs with BLAST sequence similarities to genes that had established annotations and which also had a perceived relevance to reproduction in vertebrates. In the cases where several ESTs showed similar BLAST sequence similarities, usually only one EST was selected for QPCR analysis meant to serve as a proxy validation for the larger EST group (Table 1). The relative differential expression between male and female *T. thynnus* gonads was assessed for a total of 13 transcripts. Nine transcripts were chosen to represent gene categories of interest with significant higher expression in the ovaries including: ZPC1-TTC00305, vitelline envelope protein gamma-TTC00056, alveolin-TTC00935, choriogenin L-TTC04136 (egg envelope formation); Cathepsin S-TTC00230 (yolk proteolysis); Aquaporin 1-TTC00180, Tmc6 related protein 1-TTC04625 (oocyte hydration); Fatty acid-binding protein, adipocyte-TTC00964, Epididymal secretory protein E1 precursor-TTC00209 (lipid accumulation) (Figure 3). Four transcripts were chosen to represent the gene categories of interest with significantly higher expression in the testis including: Synaptonemal complex protein 3-TTC02745 (meiosis); T-complex-associated testis-expressed protein 1-TTC02749 (sperm motility); Brain-type fatty acid binding protein-TTC05153, Intestinal fatty acid-binding protein-TTC05128 (lipid metabolism) (Figure 3).

**Table 1:** **A subset of significant differentially expressed ESTs from *T. thynnus* female and male gonad tissue**

***T. thynnus*EST identifier**	***T. thynnus*EST accession number**	**Relative fold change**	**Blast2GO sequence similarity description**	**Reference sequence**	E-value^*^
**Significantly differentially expressed at greater abundances in*T. thynnus*ovarian tissue relative to testes tissue**					
TTC00054	EC091690	6.1	ZPC1 [*Oryzias latipes*]	AAN31188.1	1e-26
TTC00677	EC092378	5.2	ZPC1 [*Cynoglossus semilaevis*]	ABY81291.1	7e-21
TTC00305	EC091965	4.5	ZPC1 [*Cynoglossus semilaevis*]	ABY81291.1	1e-22
TTC00023	EC091658	4.2	ZPC1 [*Cynoglossus semilaevis*]	ABY81291.1	2e-73
TTC05527	EH000098	2.6	ZPC1 [*Oryzias latipes*]	AAN31188.1	2e-24
TTC00911	EC092645	4.1	zona pellucida sperm-binding protein 4 precursor [*Felis catus*]	NP_001009260.1	8e-12
TTC00906	EC092640	4.7	ZPB domain containing protein [*Oryzias latipes*]	NP_001098217.1	8e-51
TTC00944	EC092682	4.2	ZP1 precursor [*Mus musculus*]	AAC48480.1	1e-12
TTC03982	EG630106	5.8	ZPB [*Oryzias latipes*]	AAN31187.1	8e-61
TTC00652	EC092350	3.2	ZP2 [*Cyprinus carpio*]	CAA96572.1	2e-08
TTC04154	EG630316	8.6	ZPC1 [*Cynoglossus semilaevis*]	ABY81291.1	7e-52
TTC04773	EG631058	3.3	ZPC2 [*Oryzias latipes*]	AAN31189.1	1e-28
TTC00418	EC092092	3.1	ZPC1 [*Cynoglossus semilaevis*]	ABY81291.1	9e-15
TTC04501	EG630741	2.8	ZPC1 [*Cynoglossus semilaevis*]	ABY81291.1	2e-17
TTC00056	EC091692	6.4	vitelline envelope protein gamma [*Oncorhynchus mykiss*]	NP_001117746.1	3e-09
TTC00654	EC092352	4.2	ZP3 [*Carassius auratus*]	CAA88838.1	1e-14
TTC00493	EC092174	7.6	egg envelope glycoprotein [*Xenopus laevis*]	AAY22122.1	4e-05
TTC01165	EC092929	3.5	egg envelope glycoprotein [*Xenopus laevis*]	AAY22123.1	1e-07
TTC04306	EG630505	3.0	egg envelope glycoprotein [*Xenopus laevis*]	AAY22122.1	3e-10
TTC00935	EC092671	4.2	alveolin [*Oryzias latipes*]	NP_001098139.1	2e-12
TTC04136	EG630294	2.9	choriogenin L [*Fundulus heteroclitus*]	BAJ07538.1	1e-04
TTC00085	EC091723	10.3	Cathepsin Z precursor [*Osmerus mordax*]	ACO09238.1	8e-51
TTC01243	EC093018	3.9	cathepsin Z-like protein [*Lutjanus argentimaculatus*]	ACO82387.1	4e-24
TTC00340	EC092003	2.4	Cathepsin Z precursor [*Osmerus mordax*]	ACO09238.1	6e-85
TTC00230	EC091881	5.5	cathepsin S [*Oryzias latipes*]	NP_001098157.1	2e-97
TTC04658	EG630923	5.2	cathepsin S [*Oryzias latipes*]	NP_001098157.1	5e-42
TTC00689	EC092390	4.9	cathepsin S [*Ictalurus punctatus*]	ABD65539.1	3e-18
TTC00766	EC092478	4.8	cathepsin S [*Oryzias latipes*]	NP_001098157.1	7e-32
TTC04399	EG630616	4.7	cathepsin S [*Ictalurus furcatus*]	ADO27765.1	9e-13
TTC00605	EC092298	4.5	cathepsin K [*Fundulus heteroclitus*]	AAO64475.1	2e-13
TTC04464	EG630693	4.2	cathepsin S [*Oryzias latipes*]	NP_001098157.1	5e-43
TTC03943	EG630062	2.0	cathepsin S precursor [*Fundulus heteroclitus*]	AAO64477.1	1e-06
TTC00346	EC092009	4.8	cathepsin S [*Oryzias latipes*]	NP_001098157.1	3e-28
TTC00235	EC091886	3.8	cathepsin [*Paralabidochromis chilotes*]	AAQ01147.1	6e-15
TTC06758	EH667736	3.0	cathepsin L-like protein [*Lutjanus argentimaculatus*]	ACO82386.1	4e-50
TTC04596	EG630853	3.8	cathepsin K precursor	NP_031828.2	3e-28
TTC04425	EG630647	5.7	aquaporin [*Solea senegalensis*]	AAV34612.1	1e-21
TTC04368	EG630581	5.2	aquaporin 1o [*Cynoglossus semilaevis*]	ADG21867.1	2e-40
TTC00180	EC091829	4.5	aquaporin 1o [*Cynoglossus semilaevis*]	ADG21867.1	2e-34
TTC00584	EC092275	5.0	aquaporin [*Solea senegalensis*]	AAV34612.1	3e-11
TTC02704	EC918066	4.5	aquaporin 1o [*Cynoglossus semilaevis*]	ADG21867.1	2e-19
TTC04625	EG630885	23.1	Tmc6-related protein 1 [*Takifugu rubripes*]	AAP78785.1	4e-07
TTC01498	EC421545	9.6	fatty acid binding protein 11b [*Danio rerio*]	NP_001018394.1	3e-20
TTC00750	EC092461	4.7	fatty acid-binding protein liver-type [*Ictalurus punctatus*]	ADO29352.1	6e-34
TTC04564	EG630815	4.7	fatty acid-binding protein liver-type [*Ictalurus punctatus*]	ADO29352.1	2e-27
TTC07800	EL610584	4.4	Fatty acid-binding protein, heart [*Anoplopoma fimbria*]	ACQ57957.1	5e-43
TTC04356	EG630567	4.0	fatty acid-binding protein liver-type [*Ictalurus punctatus*]	ADO29352.1	1e-26
TTC03876	EG629978	3.5	fatty acid binding protein 1 [*Mesocricetus auratus*]	AAV33399.1	4e-07
TTC00964	EC092703	2.8	Fatty acid-binding protein, adipocyte [*Salmo salar*]	NP_001134675.1	5e-54
TTC00209	EC091860	7.6	Epididymal secretory protein E1 precursor [A. fimbria]	ACQ58497.1	2e-32
TTC04551	EG630798	7.2	Epididymal secretory protein E1 precursor [*Osmerus mordax*]	ACO09051.1	2e-38
TTC00127	EC091768	6.2	Epididymal secretory protein E1 precursor [A. fimbria]	ACQ58497.1	5e-38
TTC01068	EC092821	3.7	Epididymal secretory protein E1 precursor [*A. fimbria*]	ACQ58497.1	2e-13
TTC00421	EC092095	2.8	Epididymal secretory protein E1 precursor [*A. fimbria*]	ACQ58497.1	2e-30
**Significantly differentially expressed at greater abundances in*T. thynnus*testes tissue relative to ovarian tissue**					
TTC05519	EH000090	18.2	tctex1 domain-containing protein 1-A [*Xenopus laevis*]	NP_001090117.1	1e-28
TTC05755	EH000358	11.6	tctex1 domain-containing protein 1-B [*Xenopus laevis*]	NP_001106901.1	2e-25
TTC02749	EC918118	12.6	T-complex-associated testis-expressed protein 1 [*R.norvegicus*]	NP_001101676.1	2e-42
TTC02745	EC918114	12.4	synaptonemal complex protein 3 [*Oncorhynchus mykiss*]	NP_001117979.1	4e-39
TTC05128	EG999641	6.6	intestinal fatty acid-binding protein [*Paralichthys olivaceus*]	ABV91589.1	5e-12
TTC05153	EG999669	33.9	brain-type fatty acid binding protein [*Oryzias latipes*]	NP_001116389.1	8e-11

^*^ Expect value is the likelihood that the sequence similarity match is a random occurrence on the given database.

## Discussion

Bluefin tunas are apex oceanic predators found in the Atlantic, Southern and Pacific oceans. They are an important commercial resource that are in decline primarily due to over-fishing. Effective management of the wild resources as well as the success of bluefin tuna aquaculture will rely heavily on a thorough understanding of the reproductive biology of these fishes. This investigation was initiated to further our knowledge of bluefin tuna reproductive biology by using a contemporary transcriptome approach novel to bluefin tuna research. The transcriptional expression profiles from *T. Thynnus* male and female mature gonad tissues and their transcript constituents are discussed here with relation to their importance to the reproductive processes of bluefin tuna. ESTs of interest that were over expressed in the *T. thynnus* ovarian tissue are discussed first followed by those that were significantly more highly expressed in the testis tissue. The results from this investigation are likely to be applicable for the *Thunnus* genus as a whole due to the relatively low genomic variation among its members. This is because the Genus is of relatively recent origin as evidenced by comparatively low levels of inter-specific nucleotide variation among member species, ranging from 0.01 for the mitochondrial CO1 gene to 0.03 for nuclear non-coding ITS-1 sequences [24]. Furthermore, because nuclear genomes mutate more slowly than mitochondrial genomes, and because nonsynonymous sites are much less free to vary than non-coding regions [25], we believe our transcriptional methods for Atlantic bluefin will be applicable to other Thunnus species.

### EST differential expression in ovarian tissue

Reproductive strategies in fish vary greatly including aspects such as attraction, gonochorism and sex change, synchronous and asynchronous ovarian development, spawning temporal and spatial patterns and parental care [26,27]. However among teleosts the fertilization strategy is dominated by an oviparous regime in which oocyte development leading up to external fertilization in teleosts appears to be a uniform process. This coordinated assembly of the fish egg is classified into six main sequential phases: oogenesis, primary oocyte growth, cortical alveolus stage, vitellogenesis, maturation and ovulation [28,29].

The histological examination of the *T. thynnus* ovarian tissues used for this investigation showed that oocyte development had reached a mature stage when the fish were captured and sampled. This observation is in concordance with previous findings whereby large bluefin tuna are considered to be present in the Gulf of Mexico predominately from March to June for spawning [30]. The microarray and QPCR results generated from this study using the same *T. thynnus* ovarian samples provides a transcriptome profile of these mature individuals. This permits examining the portion of the transcriptome that may be utilized to generate a gender specific maturation profile. In this regard we detected differential expression of a number *T. thynnus* ESTs present on the BFT 4X44K array that were homologous with annotated genes consistent with mature oocyte presence. Specifically, *T. thynnus* ovarian differentially expressed transcripts pertaining to oogenesis related gene categories to be discussed herein will include those concerned with egg envelope formation, yolk proteolysis, oocyte hydration, and lipid accumulation.

The egg envelope often termed the vitelline envelope (VE) in teleosts is involved in processes including fertilization and protection of the egg and embryo [31]. The VE is formed during oocyte development between the follicle cells and plasma membrane of the oocyte [32] and is composed of a relatively thick proteinaceous extracellular matrix usually of a few major glycoproteins [33]. The terminology for VE proteins and genes is broad due in part to different names being ascribed to various vertebrate groups, including zona pellucida, vitelline membrane, chorion, egg shell protein, zona radiata and vitelline envelope. However, amino acid sequence homologies among these proteins aid in characterizing them via the typical presence of a specific zona pellucida like domain (ZP) [32].

Microarray analysis revealed a total of 21 *T. thynnus* ESTs with significant sequence similarities to genes encoding VE proteins (Table 1). These transcripts were detected at greater abundance in the female gonad of *T. thynnus*. QPCR analyses of a subset of these VE gene homologs (ZPC1-TTC00305; vitelline envelope protein gamma-TTC00056; alveolin-TTC00935; choriogenin L-TTC04136) validated the differential expression shown by microarray analysis (Figure 3). While these ESTs’ identity and function as VE protein genes is inferred from their sequence similarities it should be noted that previous studies identifying the VE proteins of teleosts have routinely identified only a few major proteins and genes associated with the VE [33-38]. This trend is perhaps an outcome from the ‘protein-down’ approach often employed in these studies including techniques whereby VE gene sequences were searched for using degenerative PCR primers designed from the amino acid sequences of the purified VE proteins. More recently, with the proliferation of genome sequencing among organisms, specifically teleost species zebrafish and medaka, has been established that both these fish have multiple gene isoforms containing ZP domains [37,39]. These findings help to explain the similar detection of multiple ESTs containing ZP domains preferentially expressed in the mature ovaries of *T. thynnus* (Table 1).

Hydration of the oocyte is an integrated process, essential to the maturation of pelagophil eggs [40]. During the final stages of oocyte maturation, an osmotic gradient is created between the oocyte and the interstitial fluid or oviduct leading to a rapid swelling of the oocyte. Hyperosmolality of the yolk drives the oocyte hydration due in part to the proteolytic cleavage of oocyte yolk proteins sequestered during vitellogenesis. This protein degradation results in a rapid increase in concentration of free amino acids (FAA) and small peptides. However, yolk hydrolysis is not the only mechanism driving oocyte hydration. The accumulation of inorganic ions, particularly Cl^-^ in the oocyte is also contributing to the hydration [41]. The rise in these molecules (FAA and inorganic ions) helps to build an osmotic potential between the oocyte and the surrounding environment. This gradient, in conjunction with the water channel protein, aquaporin, drive the hydration of the oocyte which is essential for the production of pelagophil buoyant eggs. Differential expression of transcripts in maturing ovarian tissue of *T. thynnus* with sequence similarities to genes putatively involved in these processes are discussed here together. Namely, protease-encoding genes from the cathepsin family as well as the transmembrane channel-like protein 6 (TMC6) and aquaporin genes are considered.

Cathepsin proteases have been reported to be responsible for yolk protein hydrolysis in teleosts [42-45]. Significant sequence similarities with cathepsin coding genes of 15 *T. thynnus* transcripts that were differentially expressed in the maturing ovarian tissue of *T. thynnus* in this investigation indicate a similar putative function for these ESTs (Table 1). However which members of the cathepsin family are responsible for the final stages of yolk protein hydrolysis is unclear. Cathepsin L [43] and Cathepsin B [44,46], have both been purported separately as the main protease responsible for the final hydrolysis of the yolk proteins in different teleost species. Furthermore, cathepsin D has been reported as the initial protease responsible for cleaving the yolk precursor protein, vitellogenin, into the yolk proteins [43,47]. Interestingly, none of these cathepsins seem likely to be involved in yolk proteolysis in *T. thynnus* maturing oocytes. In this investigation only one of the 15 *T. thynnus* transcripts preferentially expressed in maturing ovarian tissue showed a sequence similarity to cathepsin L while the remaining EST sequences aligned with cathepsin Z precursor (3) and cathepsin S (14) (Table 1). The potential that the cathepsins putatively involved in yolk proteolysis were not detected in this investigation due to the *T. thynnus* ovarian tissues used being at a different oocyte developmental stage is unlikely. Raldua et al. [44] showed that temporal expression of these yolk proteolysis enzymes is detectable from early vitellogenesis and are stored in the egg as latent acid-activatable proenzymes. Histological examination of the ovarian tissues used in this study indicates female specimens (3 of 4) are in late vitellogenesis during which yolk proteolysis enzymes should be expressed. Therefore it seems likely that *T. thynnus* employs different cathepsin proteases for the initial cleavage of the vitellogenin as well as the final yolk proteolysis in comparison to the current reported enzymes for teleost species.

Although yolk proteolysis has been accounted for an increase in oocyte osmolality, the accumulation of inorganic ions is thought to provide approximately half of the osmolytes [41]. The mechanism by which these inorganic ions accumulate in pelagophil oocytes is largely unknown. *T. thynnus* – trans-membrane channel protein 6 (TMC6) may function to transiently accumulate inorganic ions, specifically Cl^-^ ions into the oocyte thus raising the osmolality required for full oocyte hydration. This transcript was expressed 23 times higher in the ovaries compared to the testes of *T. thynnus* and had the greatest differential expression profile of all 1273 ESTs highlighted in the microarray analysis (Table 1). Although currently no annotation references for TMC6 to egg hydration are available, we note that TMC6 is a part of a larger transmembrane channel family. While the discovery of this gene family is relatively novel, a consensus is building in the literature that these proteins may function as ion channels, pumps or transporters [48-50]. Specifically, Hahn et al. [48] concluded that TMC6 is likely to have Cl^-^ channel activity based on sequence homology. Considering the ovarian specific expression of *T. thynnus Tmc6* and its homology-based annotation linking it to Cl^-^ channelling, we propose that TMC6 in the *T. thynnus* oocyte is involved in the oocyte hydration via the accumulation of Cl^-^ in the oocyte thus raising the internal osmolality and driving the influx of water through aquaporin channels.

Following yolk proteolysis and accumulation of inorganic ions, teleost ooycte hydration is typically achieved by an influx of water across an osmotic gradient mediated by the water channel protein aquaporin [41]. Five transcripts differentially expressed in *T. thynnus* ovarian tissue showed sequence similarities to aquaporin suggesting a similar mechanism may be employed in *T. thynnus* (Table 1). Although oocyte hydration occurs just prior to spawning, the expression of the aquaporin protein begins during early vitellogenesis in a similar manner to yolk proteases and is stored within the egg until they are activated by an unknown mechanism [40].

Teleost ovarian development is a nutrient demanding process of which lipids are a substantial requirement for oocyte development. The lipids necessary for developing oocytes are mobilized from reserves within the animals including the liver, muscle and other tissues. Considering the importance of lipid transport and accumulation, differential expression of transcripts with sequence similarities to genes encoding lipid transport proteins in *T. thynnus* ovarian tissue reported in this study are discussed.

A vital component facilitating cellular lipid transport among others is the family of fatty acid binding proteins (FABP). FABPs are cytoplasmic proteins whose primary role is to regulate fatty acid uptake and intracellular transport [51]. Seven differentially expressed transcripts (TTC01498; TTC00750; TTC04564; TTC07800; TTC04356; TTC03876; TTC00964) in ovarian tissue of *T. thynnus* show sequence similarities with the *Fabp* gene family, particularly *Fabp1* and *Fabp4* (Table 1). Differential expression for TTC00964 was confirmed with QPCR analyses (Figure 3). Considering the intracellular nature of FABP we propose that the ovarian specific expression of these seven transcripts in *T. thynnus* maturing ovaries is likely involved in the membrane trafficking and sequestration of lipids in the oocytes which is a requirement for normal embryo development. This assertion is supported by observations that expression of both *Fabp1* and *Fabp4* are well documented in adipose tissue and liver, both of which are heavily involved in lipid sequestration [52,53]. An additional function for *Fabp* homologues expressed in *T. thynnus* ovarian tissue beyond oocyte lipid accumulation is indicated by a significant sequence similarity between transcripts TTC01498 and *FABP11*. Agulleiro et al. [54] observed in a teleost fish (*Solea senegalensis*) that *Fabp11* (restricted to fishes) was expressed in ovarian follicle cells positively correlated with ovarian atresia (reabsorption of the oocyte), particularly postovulatory regression. While our histological examination of subsamples from the *T. thynnus* ovarian tissue used for microarray analysis was not consistent with postovulatory regression some minor oocyte atresia was observed. Minor oocyte atresia is known to occur normally during ovarian development. The differential expression of TTC01498 in ovarian tissue undergoing minor atresia used for this investigation and the observations of Agulleiro et al. [54] support a putative role for FABP11 in fatty acid trafficking specifically related to oocyte atresia.

Another lipid transport gene of interest to this study is Epididymal secretory protein E1 gene. Despite its title being suggestive of a testicular function, five *T. thynnus* ESTs bearing a significant sequence resemblance to this gene were observed to be preferentially expressed in the ovary in comparison to the testes of *T. thynnus* (Table 1). The relative expression of one of the transcripts (TTC00209) was further examined with QPCR confirming the microarray analysis for this EST and serves as a proxy for the remaining four ESTs (Figure 3). The alternative name for Epididymal secretory protein E1 is and Niemann-Pick disease type C2 (*Npc2*). When considering this alternative gene name and protein functions, its expression in *T. thynnus* ovaries seems more plausible. This protein is known to be involved in cholesterol homeostasis, specifically intracellular cholesterol trafficking [55]. It functions such that after lipoproteins carrying cholesterol are endocytosed and hydrolyzed in lysosomes, the NPC2 is responsible for their exit from the lysosome to where required [56]. This cholesterol homeostatic function likely explains the higher ovarian transcript abundance for the seven *Npc2*-like *T. thynnus* ESTs, given the high lipid requirement for fish oocyte maturation, of which cholesterol is a significant component. Greater concentration of these *Npc2*-like transcripts expressed in the maturing ovary in comparison to the testes of *T. thynnus* would be necessary for the NPC2 to process the influx of cholesterol via intercellular lipid transport proteins like low density lipoprotein (LDL) [57].

### EST differential expression in the testis tissue

Although the development of eggs and sperm share common principles, many aspects of gametogenesis differ between the sexes. Knowledge on spermatogenesis in fish is limited to a few species used in basic research and/or aquaculture biotechnology [58]. The microarray and QPCR results generated from this study using mature *T. thynnus* testes tissue has helped to identify a number of these differences on a molecular level. Specifically, *T. thynnus* testes differentially expressed transcripts related to spermatogenesis is discussed including transcripts potentially involved in meiosis, sperm motility and lipid metabolism.

*T. thynnus* testes are described as having unrestricted spermatogonial distribution whereby gametes are synchronously produced in cysts spread throughout the germinal compartment [59]. Histological classification of the *T. thynnus* testes tissues used in this study (Figure 1) was adapted from [23] classification system for yellowfin tuna. These cysts are present at all spermatogenesis stages during testicular maturation. Final sexual maturation in *T. thynnus* involves a significant enlargement of the testes, but unlike females no apparent noteworthy histological changes are present, with the exception of a marginally higher frequency of the most advanced stages of spermatogenesis in fully mature bluefin tuna [59]. Histological analysis of subsamples from the testes tissue used in this investigation shows spermatozoa are present which is the final product in mature tuna.

The synaptonemal complex (SC) is a meiosis specific structure formed during the first meiotic prophase of germ cells within sexually reproducing organisms. The SC is made up of three proteins - synaptonemal complex protein 1, 2 and 3 (SYCP), involved in chromosome pairing and recombination [60]. *T. thynnus* EST TTC02745 exhibits a significant sequence similarity to *Sycp3* and is shown to be highly differentially expressed in the mature testis of the *T. thynnus* in comparison to ovaries (Table 1, Figure 3). SYCP3 is the best characterized of all the SC proteins albeit studied predominately in mammals. However, recent investigations characterizing this protein in fish are highlighting some divergences with the mammalian SC protein [61,62]. Based on previous SCYP3 annotations this protein is not considered to be sexually dimorphic, yet our expression analysis indicated the contrary for *Sycp3*-like EST TTC02745. This is an interesting observation when considering the spawning modes of the species. The testis development of *T. thynnus* is reported as that of the unrestricted spermatogonial testicular type whereby continuous spermatogenesis occurs in the testes tubules [59]. Thus the testes of *T. thynnus* contain germ cells at all stages of development including the first stages of meiosis during which SYCP3 is known to be expressed.

*T. thynnus* females have been described as serial spawners characterized by asynchronous ovarian development in which all stages of oogenesis are continuously present during the spawning season [63]. Despite this apparent continuous gametogenesis similarity, *Sycp3* in ovarian tissue is expressed at a reduced level in comparison to testes tissue. Mammalian studies explain this disparity in that the first stages of meiosis in female germ cells occur during embryonic development after which they go into meiotic arrest until meiosis resumption at puberty [64]. Males in contrast do not begin meiosis until puberty. This meiotic arrest explanation relies on an assumption that female fish like mammals have a finite number of germ cells that cannot be replenished or regenerated. However, this theory has not been established outside mammals and conversely there is some evidence that highly fecund lower vertebrates may produce new oocytes from mitotic oogonia [65]. Therefore considering that *T. thynnus* is a highly fecund species in conjunction with potentially continuously replenishing oocytes, the relatively low meiosis specific *Sycp3*-like EST ovarian expression in comparison to males may indicate some additional mechanism of SYCP3 sexual dimorphism beyond the established mammalian temporal patterns. Furthermore, potential indications for a sex specific role for SYCP3 have been reported in mouse knock-out studies. Male mice lacking *Sycp3* expression were rendered completely sterile while females were only marginally affected remaining largely fertile [66]. This disparity in fertility mediated by SYCP3 indicates that this protein may function differently between sexes with a particular importance for male fertility.

An important molecular component of spermatogenesis is the t-complex, a chromosomal region containing genes known to specifically influence male fertility [67]. *T. thynnus* ESTs possessing sequence similarities to two genes known to map to this t-complex, *Tctex1* (TTC05519; TTC05755) and *Tcte1* (TTC02749) genes were found to be highly differential expressed in the mature testis of the *T. thynnus* (Table 1). Differential expression was confirmed with QPCR for EST TTC02749 (Figure 3). TCTE1 is considered to be involved in the species specific molecular interaction between the sperm and egg zona pellucida permitting the penetration of the sperm and fertilization [68]. Species specific recognition of gametes is particularly relevant for *T. thynnus* along with other marine spawning fish as they release their gametes in a highly dynamic environment whereby cross-fertilization is undoubtedly an issue. The molecular mechanism by which this sperm-egg recognition is achieved is largely unknown for marine fish. However the relative differential expression of *Tcte1*-like EST TTC02749 in the mature testis of *T. thynnus* suggests a role in facilitating such species specific gamete fertilization. Also part of the t-complex, TCTEX1 has also been assessed as essential for male fertility in the animals studied. However unlike *Tcte1*, null mutations of *Tctex1* affect the phenotypic function of sperm. TCTEX1 has been characterized as a cytoplasmic dynein light chain subunit involved in ubiquitous intracellular transport processes and the proper attachment between the sperm nucleus and flagellar basal body [69]. Surprisingly, Li et al. [69] found that TCTEX1 contributions to the essential cytoplasmic dynein functions are dispensable while male fertility functions were not. This was demonstrated in drosophila whereby *Tctex1* null mutants were deemed largely viable other than for complete male sterility. This further exemplifies the male specific influence that genes in the t-complex have on spermatid production. Considering ESTs TTC05519 and TTC05755 sequence similarities with *Tctex1* and their differential testis expression in *T. thynnus*, we propose these transcripts are likely to perform a similar function to that of TCTEX1 and as such are a notable aspect of the differentiation between the testis and ovarian transcriptomes of *T. thynnus*.

As previously described in the ovarian component of this discussion, fatty acid binding proteins are part of a multigene family responsible for a diverse array of functions centered on cytoplasmic fatty acid binding. The testis differential expression of transcripts TTC05153 and TTC05128 exhibiting sequence similarities to that of the brain type (*Fabp7*) and intestinal type (*Fabp2*) fatty acid binding proteins respectively is further evidence of the diversity of this gene family (Table 1). Differential expression of these FABP-like ESTs is discussed below with reference to *T. thynnus* gonads and their possible function.

Much of FABP7 characterization thus far has largely focused on its expression in the brain of vertebrates, highlighting its association with essential highly polyunsaturated long-chain fatty acids present there, particularly docosahexaenoic acid (DHA) [70]. FABP7 has been shown to have the highest affinity for DHA among all the FABP [71]. Although little is known regarding FABP7 function in the testis, similarities in the fatty acid profiles between the two tissues suggests an explanation for the expression of *Fabp7*-like transcript TTC05153 in the testis of *T. thynnus*. Like the brain, DHA is also present at high concentrations in the retinitis and in mature testis (sperm tail) of vertebrates [72]. Apart from the presence of DHA an additional similarity between these tissues is the presence of axonemes (organelles composed of microtubules). DHA is theorized to contribute to membrane fluidity necessary for the motility of the axoneme [73]. This link is further supported in that DHA deficiencies have been noted to cause retina pigmentosa as well as sperm abnormalities [72]. Taken together we propose that TTC05153 functions similarly to *Fabp7* and its expression in the mature testis of *T. thynnus* is involved in DHA intracellular transport necessary for sperm motility. However it should be noted that this hypothesis does not adequately explain how DHA is incorporated into oocytes. DHA is a well known essential fatty acid present in marine fish eggs, required during embryogenesis particularly for eye and brain development [28]. It may be that the large difference in relative expression of *Fabp7*-like EST TTC05153 between the mature testes and ovaries from *T. thynnus* is simply due to a sexually dimorphic requirement for DHA; oocyte requirements for DHA while significant may be met with considerably less than that of sperm.

Similar to FABP7, much of the previous characterization efforts related to FABP2 function have involved tissues other than testes. Specifically, functional investigations for FABP2 have focused on the intestinal tissues, noting specific polymorphisms in this genes sequence are correlated with obesity and insulin resistance in vertebrates. This has lead to the hypothesis that FABP2 is involved in the transmembrane uptake of dietary fatty acids [74-76]. However, gene knock-out studies in mice showed that FABP2 is not essential to dietary fat absorption but may instead function as a lipid-sensing component of energy homeostasis that alters energy balance and thus body mass in a gender-specific fashion [77]. While this hypothesis is derived from the intestinal studies, a gender-specific role may explain the differential expression of *Fabp*-like EST TTC05123 in the testis of mature *T. thynnus* (Figure 3). Sex-specific energy budgets are a well established concept owing to different reproductive requirements, especially in highly migratory pelagic fish [78-80]. Therefore based on *Fabp2*-like EST TTC05123 observed sexual dimorphism in expression and the established gender-specific energy requirements for fish, we propose *Fabp2*-like EST TTC05123 is involved in energy homeostasis. Furthermore we suggest that the greater expression of *Fabp2*-like EST TTC05123 in testis tissue compared to ovarian tissue may indicate that male *T. thynnus* are using and/or mobilizing a greater proportion of lipids for energy homeostasis than females who are presumed while present in spawning locations such as the Gulf of Mexico to be sequestering their lipid reserves for oocyte development [81].

## Conclusions

In summary, the transcriptomic approach is a useful tool for examining differential gene expression profiles for the gonadal tissue of *T. thynnus*. In this study, 7068 transcripts were assessed for their differential gene expression in testis and ovarian tissues of sexually mature *T. thynnus*. ESTs bearing sequence similarities to annotated genes and that were significantly differentially over-expressed between the two tissues were considered with respect to their organ’s primary gametogenic functions. A number of important components of oogenesis were discussed in relation to identified ESTs including egg envelope formation, yolk proteolysis, oocyte hydration, and lipid metabolism. Similarly, ESTs potentially related to components significant to spermatogenesis were also discussed, including meiosis, sperm motility and lipid metabolism. These ESTs were characterized with reference to their potential role in conventional gametogenesis and where appropriate, alternative functions were proposed. Additional research is required to corroborate the hypothesized functions of the ESTs identified herein in *T. thynnus* gametogenesis. Although Atlantic bluefin reproductive research is a challenging undertaking with tissues difficult to obtain, further transcriptome styled investigation of other tissues taken in a non-destructive method may provide valuable insight and proxy indications for bluefin reproductive condition. The specific ESTs identified herein are important as potential biomarkers for reproductive development, gender distinction and maturation. Such information and non-destructive tools would be highly desirable for both fisheries management and aquaculture development of the Atlantic bluefin by allowing managers to make informed decisions on sexual maturity with little impact to these already over-exploited and highly prized animals. This investigation is the first application of microarray technology for bluefin tunas and has demonstrated the efficacy by which this technique may be used for further characterization of the many unknown biological aspects for this valuable fish.

## Methods

### Animal and tissue collection

Gonad tissue samples were collected from mature *T. thynnus* by observers as part of the Pelagic Longline Observer Program coordinated by the National Oceanic and Atmospheric Administration (NOAA). Observers stationed aboard commercial longline vessels in the Gulf of Mexico during May of 2009 sampled tissues from *T. thynnus* taken as bycatch on pelagic longlines within 30 minutes of death. Where possible, curved fork length of captured *T. thynnus* were recorded. Gender was determined by gross dissection and review of gonad histology. Gonad tissue was sampled from the fish in a sterile and RNAse free manner and fixed immediately in RNA*later*® (Applied Biosystems, Foster City, CA, USA) for gene expression analyses and 10% phosphate buffered formalin for subsequent histological processing. A 1:10 sample to fixative volume ratio was used for both fixatives. Tissues for RNA analysis were immediately stored at 4°C overnight and then transferred to −20°C until processing.

### Histology

Histological sections were sliced from formalin preserved and paraffin embedded gonad samples by IDEXX Laboratories (Sacramento, CA, USA) and stained with with haematoxylin and eosin. Sexual maturity and spawning status were determined histologically according to the methods used by Schaefer [23] and Itano et al. [22].

### RNA isolation

Tissue samples were homogenized with a TissueLyser II and stainless steel beads (Qiagen, Valencia, CA, USA). Total RNA was purified from gonad samples fixed in RNA*later*® using TRIZOL® reagent as recommended by the manufacturer (Invitrogen Life Technologies, Carlsbad, CA, USA). Concentration and purity of the RNA were determined using a spectrophotometer (NanoDrop® ND-1000, NanoDrop Technologies Inc., Wilmington, DE, USA) with 230, 260 and 280 nm readings. RNA quality was assessed for all samples by visualization on a denaturing formaldehyde RNA gel (protocol recommended by Qiagen, Valencia, CA, USA) and ethidium bromide staining.

### Microarray

#### Microarray platform description

The microarray platform used during this investigation for the purposes of gonad transcriptome expression profiling is entitled BFT 4X44K Array. The platform is a custom commercial Agilent Technologies 4 X 44K format (Agilent Technologies, Santa Clara, CA, USA), capable of the independent hybridization of four separate microarrays per microarray chip. Each independent microarray contains 44 000 *in situ* synthesized oligonucleotide probes with an average length of 50 nucleotides. The probes represent published ESTs derived from Atlantic bluefin tuna liver, ovaries and testis [18]. A total of 10163 ESTs were downloaded from GenBank (accession numbers: EC091633-EC093160, EG629962-EG631176, EC917676-EC919417, EG999340-EG999999, EH000001-EH000505, EH667253-EH668984, and EL610526-EL611807) using the Trace2bdEST component of the PartiGene EST-software pipeline [82]. These ESTs were clustered using TIGR Gene Indices Clustering software to create a non-redundant set of ESTs [83]. A total of 7068 EST clusters were generated and annotated with the Blastx algorithm [84] and Blast2GO software suite [85]. Array Designer software (Premier Biosoft International, Palo Alto, CA, USA) was used to select from the 7068 ESTs approximately three sense strand probes per EST each being a unique complimentary sequence for that EST and approximately 50 oligonucleotides in length. These probes were all duplicated on the microarray to a combined total of 44 000 probes per array. The BFT 4X44K Array platform design is publically accessible at the Gene Expression Omnibus (GEO) repository ( http://www.ncbi.nlm.nih.gov/geo/), accession number GPL10007.

#### Microarray experimental design, target preparation and hybridization

A total of eight *T. thynnus* gonad specimens were utilized for the microarray hybridizations, four females and four males representing biological replicates for both sexes. Total RNA was extracted individually from each of these samples as previously described. Aliquots of the extracted total RNA samples were taken and pooled equally among the eight individuals according to RNA concentration. This pooled RNA extraction was used as a common reference to compare variations in mRNA expression among the eight gonad samples. Each gonad sample was individually hybridized against the reference sample in a two-color design, totalling eight microarrays that were hybridized for the investigation in this manner. Target preparation including reverse transcription of total RNA, RNA amplification by *in vitro* transcription and aRNA labelling, was conducted using the Amino Allyl MessageAmp^TM^ II aRNA Amplification Kit (Applied Biosystems, Foster City, CA, USA) according to manufacturer’s instruction. Briefly, reverse transcription reactions were performed using T7 Oligo(dT) primer with Arrayscript reverse transcriptase. Second strand synthesis was then performed and cDNA purified using cDNA filter cartridges and used as template for aRNA synthesis in *in vitro* transcription reactions. aRNA was then purified using aRNA filter cartridges and yield and quality of aRNA was assessed by spectrophotometer as previously described. Up to 20 μg per sample of amino allyl-modified aRNA was labeled with Cy3 or Cy5 NHS ester dyes and subsequently column purified.

Hybridization of the microarrays followed the Two-Color Microarray-Based Gene Expression Analysis protocol with reagents from the Gene Expression Hybridization Kit (Agilent Technologies, Santa Clara, CA, USA). Briefly, 825 ng each of Cy3 and Cy5 labelled aRNA was fragmented to nucleotide lengths of 60 – 200 in a fragmentation mix and then added to the hybridization buffer. The hybridization mixes were then applied separately to all four microarrays present on the BFT 4X44K Array and individually sealed with gaskets dividers within a Hybridization chamber (Agilent Technologies, Santa Clara, CA, USA). Microarrays were hybridized at 65°C for 17 hours in a rotating hybridization oven. Following hybridization, microarrays were washed according to the Two-Color Microarray-Based Gene Expression Analysis protocol (Agilent Technologies, Santa Clara, CA, USA). Briefly, the hybridisation chamber is disassembled and the microarray slide is washed for 1 minute in GE wash buffer 1 at room temperature and then transferred to GE wash buffer 2 at 37°C for an additional 1 minute. Following the second wash the slide is slowly removed from the buffer minimising droplet adherence to the slide and scanned immediately. Scanning was performed using the Axon GenePix® 4000B microarray scanner (Molecular Devices, Sunnyvale, CA, USA) at 5 μm pixel resolution with automated photomultiplier balancing used to determine signal intensity and channel balancing.

### Analysis

Each microarray on the BFT 4X44K Array slide was scanned separately with the resulting images saved as .tiff files. Feature Extraction 4.0 image analysis software (Agilent Technologies, Santa Clara, CA, USA) was used to extract and process these raw microarray images. Briefly, the Feature Extraction processing pipeline begins by placing a grid specific to the BFT 4X44K Array on the scanned image and identifying each microarray feature. Non-uniform outliers are excluded followed correction of the raw mean signal intensity values by computing background, bias and error values. Global Lowess normalization was used to correct for dye biases, before calculating the log ratios of dye-normalized signals for each feature. The microarray data is publically accessible at the Gene Expression Omnibus (GEO) repository ( http://www.ncbi.nlm.nih.gov/geo/) accession number GSE34084.

The Feature Extraction output files generated were imported into GeneSpring GX 11.0 (Agilent Technologies, Santa Clara, CA, USA) software for microarray analysis. Statistical significance for difference in gene expression between the male and female gonad samples by using an unpaired Student’s *t*-test on the processed expression ratio values. A p-value cut-off criteria of <0.05 was used in conjunction with the Benjamini-Hochberg multiple testing correction. Fold change analysis was further applied to those features that were deemed statistically significant with a cut-off of two fold difference in expression. The final list of microarray features meeting the above criteria were hierarchically clustered with the Euclidean distance metric and visualized through a heat map plot.

### Quantitative real-time PCR analysis

Quantitative real-time PCR (QPCR) was used to confirm the relative expression profiles of 13 transcripts identified as significantly differentially expressed by microarray analysis. QPCR primers were designed based on the EST sequence from which the microarray oligonucleotide probe was derived (Table 2). In preparation for QPCR cDNA generation, total RNA was treated to remove any contaminating DNA with the DNA-free^TM^ kit as per the manufacturer’s protocol (Applied Biosystems, Foster City, CA, USA). Following DNAse treatment, 5 μg of total RNA from each gonad sample was used to generate cDNA with the Superscript^TM^ III Reverse Transcriptase enzyme and random primers following the manufacturer’s protocol (Invitrogen Life Technologies, Carlsbad, CA, USA). QPCR amplification reactions were performed in triplicate for each gonad cDNA sample using a Bio-Rad, iCycler iQ, Real-Time PCR Detection System using iQ^TM^ SYBR® Green Supermix (Bio-Rad, Hercules, CA, USA) in 25 μl reaction volumes as per the manufacturer’s protocol. The thermal profile for the qPCRs consisted of an activation step at 95°C for 15 min and 40 cycles of denaturing at 94°C for 40 s, annealing at 55°C for 40 s and elongation at 72°C for 1 min. After the last amplification cycle, a melt curve procedure was performed by which the temperature was cycled from 95°C for 1 min to 55°C for 10 s and then repeated another 81 times, increasing by 0.5°C per cycle for the latter temperature, confirming the presence or absence of non-specific PCR products and primer dimer. β-actin was evaluated for its suitability as a stable transcript to be used for QPCR normalization [86]. Specifically, β-actin transcript variation between all samples ranged between 18 and 20 amplification threshold cycles. Both QPCR and microarray results showed that β-actin expression levels were independent of the sex in relation to gonads thereby validating the use of β-actin for normalization of QPCR results for this investigation. 

**Table 2:** Quantitative PCR specifics

**Putative Gene Annotation**	**Microarray identifier**	**EST Accession No.**	**Sequence (5**^**′**^**-3**^**′**^**)**^**#**^	**Amplicon length (bp)**	**Amplification Efficiency (%)**
ZPC1	TTC00305	EC091965	F – CATACCACCCTTCACCCATC	212	102
			R – GCTCCACACTAGCCCATGAT		
Vitelline egg envelope gamma	TTC00056	EC091692	F – GCTTGCATGTGTCAGGCTTA	198	94
			R – GGAGAATGGCTTGACTGCTC		
Aveolin	TTC00935	EC092671	F – GCTTCTGCTGCTCTTCGTCT	198	90
			R – AACAACACCTGAGGCAGGAC		
Choriogenin L	TTC04136	EG630294	F – GAGCAGTCAAGCCATTCTCC	230	94
			R – CGTCAATCTCAGTGGCTGAA		
Cathepsin S	TTC00230	EC091881	F – GGATCGACACTGGGAACTGT	297	92
			R – CCCTCCAGTCCAGTGATTGT		
Aquaporin 1	TTC00180	EC091829	F – CCTGTTTCGCAGTCTTGGAT	191	96
			R – GGTCGGGGTAGGAATCATTT		
Tmc6-related protein 1	TTC04625	EG630885	F – CCGGTTTCTCCTCACCAATA	135	91
			R – TTGTGCGTGACATTCCTGAT		
Fatty acid-binding protein, adipocyte	TTC00964	EC092703	F – ACTGCAATGACCGAAAGACC	175	97
			R – CCTCCTTTCCGTAGGTCCTC		
Epididymal secretory protein E1 precursor	TTC00209	EC091860	F – GCTTGATGGGATTCACCTGT	215	94
			R – CGATTATTCCCATGGACCAC		
Synaptonemal complex protein 3	TTC02745	EC918114	F – AAGAGCTGAGCGGTTCAGAG	264	91
			R – TGACCGTGGTAGTTGTTCCA		
T-complex-associated testis-expressed protein 1	TTC02749	EC918118	F – TGCTGGTGAAACACCTCTTG	208	92
			R – AGGGACAAAAGGGTGGAGTT		
Brain-type fatty acid binding protein	TTC05153	EG999669	F – CCTACACCTGATGACCGACA	212	98
			R – GCTGGGATGATTTGCTCATT		
Intestinal fatty acid-binding protein	TTC05128	EG999641	F – CGCAGCGAGAATTATGACAA	244	95
			R – AGCATGTCACCCTCCATCTC		

^#^F refers to the forward primer while R indicates the reverse primer.

Relative mRNA transcription abundances were calculated using the delta-delta Ct method [87]. Standard curves were generated from a five point serial dilution of cDNA synthesized from total RNA of a selected gonad sample. A reference cDNA preparation from the gonads of the female samples and another from the males were selected and used as the basis for the standard dilution series. The cDNA template differed depending on whether the target is predominantly considered expressed in male gonads or females. The dilution series forms a standard curve from which a linear relationship between threshold cycle (C_t_) and log_10_ of template concentration is determined. This standard curve was used as the basis for calculating/calibrating relative abundance values in the remaining samples. Amplification efficiency of the reactions was also determined from the standard curves of each primer pair (Table 2). All relative abundance values were normalized with respect to the relative abundance of β-actin pertaining to that specific gonad cDNA preparation. A Student’s t-test was used to determine whether differences in relative expression of the QPCR targeted ESTs were statistically significant among the male and female gonads of *T. thynnus* used in this investigation (Table 2).

## Competing interests

The authors declare they have no competing interests in the manuscript.

## Authors’ contributions

LG performed RNA extractions, microarray hybridizations, bioinformatics analysis, QPCR, histological analysis, interpretation of the data, drafting the manuscript and contributed to the overall experimental design and conception of the project. NJ contributed to the microarray design, microarray hybridization, QPCR and drafting of the manuscript. PC contributed to drafting of the manuscript and overall experimental design and conception. BB contributed to histological analysis, drafting of the manuscript and overall experimental design and conception. All authors read and approved the final manuscript.

## Supplementary Material

Additional file 1 ** Table listing all ESTs represented on the BFT 4X44K microarray detected as significantly differentially over-expressed in *T. thynnus* female gonad tissue in comparison to male gonad tissue.** A total of 737 ESTs are listed. Fold change from the microarray analysis is indicated as well as their putative sequence annotations and accession numbers where available. Click here for file

Additional file 2 ** Table listing all ESTs represented on the BFT 4X44K microarray detected as significantly differentially over-expressed in *T. thynnus* male gonad tissue in comparison to female gonad tissue.** A total of 536 ESTs are listed. Fold change from the microarray analysis is indicated as well as their putative sequence annotations and accession numbers where available. Click here for file
